# Manifestations of renal system involvement in hospitalized patients with COVID-19 in Saudi Arabia

**DOI:** 10.1371/journal.pone.0253036

**Published:** 2021-07-15

**Authors:** Khaled S. Allemailem, Ahmad Almatroudi, Amjad Ali Khan, Arshad H. Rahmani, Ibrahim S. Almarshad, Fahad S. Alekezem, Nagwa Hassanein, Asmaa M. El-Kady

**Affiliations:** 1 Department of Medical Laboratories, College of Applied Medical Sciences, Qassim University, Buraydah, Saudi Arabia; 2 Department of Basic Health Sciences, College of Applied Medical Sciences, Qassim University, Buraydah, Saudi Arabia; 3 Department of Laboratory and Blood Bank, King Fahd Specialist Hospital, Buraydah, Saudi Arabia; 4 Department of Clinical Pathology, Faculty of Medicine (Girls Branch), Al-Azhar University, Cairo, Egypt; 5 Department of Medical Parasitology, Faculty of Medicine, South Valley University, Qena, Egypt; University "Magna Graecia" of Catanzaro, ITALY

## Abstract

**Background:**

Although COVID-19 is an acute disease that usually resolves rapidly in most cases, the disease can be fatal and has a mortality rate of about 1% to 56%. Alveolar injury and respiratory failure are the main causes of death in patients with COVID 19. In addition, the effect of the disease on other organs is not fully understood. Renal system affection has been reported in patients with COVID 19 and is associated with a higher rate of diverse outcomes, including mortality. Therefore, in the present work, we reported the clinical characteristics and laboratory data of hospitalized patients with COVID-19 and analyzed the manifestations that indicated renal system involvement and their impact on clinical outcomes.

**Materials and methods:**

This was an observational retrospective study conducted at King Fahd Specialist Hospital, Buraydah, Saudi Arabia. All patients with COVID-19 who were admitted to this Hospital from April to December 2020 were included in the study. The patients’ findings at presentation were recorded. Demographic data and laboratory results (hematuria, proteinuria, urinary sediment cast and pus cell presence, and kidney function tests) were retrieved from electronic patient records.

**Results:**

One hundred and ninety-three patients with confirmed COVID 19 were included in the study. Dipstick examinations of all urine samples showed proteinuria and hematuria in 53.9% and 22.3% of patients, respectively, whereas microscopic examination revealed the presence of pus and brown muddy granular casts in 33.7% and 12.4% of samples, respectively. Acute kidney injury was reported in 23.3% of patients. A multivariable analysis demonstrated that hematuria was associated with acute kidney injury (AKI) (OR, 2.4; 95% CI, 1.2–4.9; *P* = 0.001), ICU admission (OR, 3.789; 95% CI, 1.913–7.505; *P =* 0.003), and mortality (OR, 8.084; 95% CI, 3.756–17.397; *P =* 0.002). Conversely, proteinuria was less significantly associated with the risk of AKI (OR, 1.56; 95% CI, 1.91–7.50; *P =* 0.003), ICU admission (OR, 2.493; 95% CI, 1.25–4.72; *P =* 0.001), and mortality (OR, 2.764; 95% CI, 1.368–5.121; *P =* 0.003). Patients with AKI had a higher probability for mortality than did those without AKI (OR, 14.208; 95% CI, 6.434–31.375; *P =* 0.003).

**Conclusion:**

The manifestations of the involvement of the renal system are not uncommon in COVID-19. These manifestations included proteinuria, hematuria, and AKI and were usually associated with a poor prognosis, including high incidences of both ICU admission and mortality.

## Introduction

Coronaviruses (CoV) are one of the most important pathogens affecting the human respiratory system. Previous epidemics of Coronaviruses included Severe Acute Respiratory Syndrome (SARS-CoV) and Middle East Respiratory Syndrome (MERS-CoV). At the end of December 2019, a new virus belonging to the beta-coronavirus 2b phylogenetic tree lineage begun a new outbreak in animals [1]. This virus is a new human-infecting coronavirus that had not previously been recognized in humans [1]. On January 30, 2020, the World Health Organization (WHO) considered the disease as a public health emergency of global concern. The WHO termed this infection coronavirus disease 2019(COVID-19), whereas the virus was named SARS-CoV-2 by the International Committee on Taxonomy of Viruses, as it was similar to SARS-CoV, which is the coronavirus responsible for SARS [2].

Angiotensin converting enzyme 2 (ACE2) has been identified as a functional receptor for SARS-CoV [3]. Studies of SARS-CoV-2 showed that the spike protein of this virus also binds to ACE2 receptors [4]. The ACE2 protein was identified in different organs, with particularly high levels in the lungs, heart, ileum, kidneys, and bladder [5]. Therefore, although the respiratory system is the main target of the SARS-CoV-2 virus, it can also affect other systems, such as the gastrointestinal tract and the renal and nervous systems [6].

SARS-CoV-2 causes an acute disease that usually resolves rapidly in most cases. Co-morbidities are observed in 20%–30% of patients with COVID-19, whereas this proportion increases to 50%–80% in patients with severe COVID-19 [7, 8]. The most common associated chronic diseases are hypertension, diabetes, cardiovascular diseases, obesity, chronic obstructive pulmonary disease, and cancer [7, 9]. Those co-morbidities are associated with an increased risk of ICU admission and death compared with the overall population [7, 9]. However, the in-hospital case fatality rate among patients with COVID-19 with these conditions ranges from 1% [10] to 56% [11].

Although alveolar injury and respiratory failure are the main causes of death in COVID-19 patients, serious involvement of multiple systems, including renal system [12], cardiovascular system [13], nervous system [14], coagulation [15] and skin [16] have been reported.

Renal involvement in COVID -19 patients may has a wide range of manifestations including hematuria, proteinuria, pyuria and acute kidney injury (AKI) [17–20]. It has been reported that proteinuria and hematuria have higher prevalence in COVID-19 than AKI [21, 22]. On the other hand, AKI has now become an alarming sign and a significant risk factor for mortality in hospitalized COVID-19 patients [7, 23, 24]. The prevalence of AKI among SARS-CoV-2 infected patients from different countries is variable [25]. Previous studies reported an incidence of AKI among COVID-19 patients ranging from 5 to 29% [8, 24, 26–31]. Presence of renal complications in COVID-19 disease is associated with higher rate of diverse outcome including mortality [22]. Previous reports of COVID-19 patients documented that hematuria, proteinuria and AKI are associated with high incidence of ICU admission, acute kidney injury and mortality [17–20, 25].

Many factors associated with SARS-CoV-2 infection may raise the risk of renal involvement in infected patients. These factors include older age, male sex, presence of co-morbidities, and pre-existing chronic kidney disease [22, 23]. Renal system involvement during SARS‐CoV‐2 infection may be explained by different mechanisms. Direct viral injury and/or disturbed hemodynamics of the kidney might cause AKI in COVID-19. In addition, the indirect effects of SARS‐CoV‐2 infection on kidney cells may result from cytokine storms, hypoxia, and drug‐associated nephrotoxicity [32]. Secondary infection with other viruses, bacteria, and fungi can contribute to AKI [32]. Sepsis was described as a probable mechanism underlying kidney injury as a consequence of altered hemodynamics [32].

A large body of articles reported the involvement of the renal system in COVID-19. Researchers also found a strong association between proteinuria, hematuria, and AKI and a poor prognosis of SARS-COV-2 infection. Therefore, for the first time, we reported the clinical characteristics and laboratory data of hospitalized patients with COVID-19 in Saudi Arabia and analyzed the prevalence of proteinuria, hematuria, pyuria, and AKI and their impact on the clinical outcomes of these patients.

## Materials and methods

The present work was an observational retrospective study that was conducted at King Fahd Specialist Hospital, Buraydah, Qassim, Saudi Arabia. All patients with COVID-19 who were admitted to that hospital from April to December 2020 were included in the study. COVID-19 infection was confirmed by SARS-CoV-2 real-time polymerase chain reaction (RT-PCR) assay in nasopharyngeal swabs. Patients were followed-up until discharge either because of recovery or death. The patients’ manifestations at the time of presentation, such as fever, cough, sore throat, headache, fatigue, muscle pain, and dyspnea, were recorded. Demographic data (age, gender, and current associated diseases (i.e., diabetes mellitus, hypertension, chronic pulmonary diseases, malignancy, chronic organ failure, etc.) and laboratory results (hematuria, proteinuria, urinary sediment cast and pus cell presence, and kidney function tests) were retrieved from electronic patient records.

The participants in the present study were classified according to the current Chinese guidelines into three groups: moderate, severe, and critical groups. Patients with clinical symptoms of fever and cough and radiographic evidence of pneumonia were defined as moderate cases. A severe case was defined as having one of the following: (i) respiratory rate >30/min, (ii) oxygen saturation ≤93%, (iii) PaO2/FiO2 ratio ≤300 mmHg, or (iv) radiographic signs of progression of pulmonary infiltration >50% in 24–48 h. A critical case was defined as having one of the following conditions: respiratory failure requiring mechanical ventilation, shock, or evidence of multiple organ failure requiring management in intensive care units [11].

AKI was defined as an increase in the serum level of creatinine (SCr) by 0.3 mg/dL at thebaseline based on kidney disease in patients with reported baseline SCr within 3 months prior to admission [33]. In patients without a previously reported SCr in the 3months prior to admission, AKI was defined as a SCr>1.2 mg/dL within the first 48 h of presentation to the emergency department, with subsequent improvement by 50% during hospitalization [22].

Proteinuria was defined by the presence of ≥1+ protein in urinalysis. Hematuria was defined as the presence of more than four red blood cells per high-power field on urine analysis. Pyuria was defined as the presence of more than 10 pus cells per high-power field on urine analysis, and urinary tract infection was defined as the presence of a positive urine culture [18, 34].

### Ethics statement

The study was conducted according to the guidelines of the Declaration of Helsinki and was approved by the Regional Research Ethics Committee, Ministry of Health, Saudi Arabia (IRB number: H-04-Q-001). Informed consent was waived since this was a retrospective study without patient identifiers.

### Statistical analysis

Data analysis was carried out using the IBM SPSS 20.0 software (SPSS Inc., Chicago, IL, USA). Continuous variables were expressed as the mean ± standard deviation. Categorical data were expressed as numbers and percentages. Comparison of two independent groups was performed using the Mann–Whitney *U* test or the chi-squared test, for continuous and categorical variables, respectively. Significance was set at *P*< 0.05.

## Results

### Baseline characteristics

One hundred and ninety-three patients with confirmed COVID-19 were included in the present study. All participants were admitted to the hospital between April and December 2020. The age of the participants ranged from 18 to 95 years (mean± SD, 64.24± 18.11 years). The majority were older than50 years (78.4%). Most patients were males (59.1%). The most common associated co-morbidities were diabetes and hypertension. The most common presenting symptom was fever. Moreover, 28.5% of the patients were critically ill patients who were admitted to the ICU for mechanical ventilation. Patients were followed-up for a duration ranging from 7 to 48 days until discharge (either for recovery or death).

According to the current Chinese guidelines, participants were classified into three groups: moderate, severe, and critical. Compared with moderate cases, critical cases had a higher mean age, a higher prevalence of co-morbid diseases among critical cases, and a high incidence of dyspnea (Table 1).

**Table 1:** Sociodemographic, laboratory, and clinical data of the patients with COVID-19.

**Variable**	**Critical COVID-19 (n = 55)**	**Severe COVID-19 (n = 126)**	**Moderate COVID-19 (n = 12)**	***P*-value**
**Clinical characteristics**				
Age (years)	71.8 (47–9)	61.75(18–95)	56.25(19–89)	0.024*
Male patients (n/%)	30 (54.5)	75 (59.5)	9 (75)	0.424
Diabetes (N/%)	36 (65.5)	52 (41.3)	7 (85.3)	0.009*
Hypertension (N/%)	21 (38.2)	40 (31.7)	3 (33.3)	0.071
Cardiac diseases (N/%)	2 (3.6)	9 (7.1)	1 (8.3)	0.636
Chronic respiratory disease	2 (3.6)	1 (0.7)	0 (0)	0.054
Malignancy	2 (3.6)	1 (0.7)	0 (0)	0.234
**Clinical presentation**				
Fever and cough (N/%)	17 (30.9)	42 (33.3)	5 (41.7)	0.276
Fever (N/%)	6 (10.9)	29 (23)	2 (16.7)	0.124
Shortness of breath (N/%)	32 (58.2)	55 (43.7)	5 (41.7)	0.002*
**Laboratory data**				
CRP (0–3.3 mg/L)	86.7 (7–201)	68.14 (1–285)	70.8 (4.5–145)	0.002*
ESR (0–29)	55.13 (16–120)	51.8 (9–112)	46.9 (13–111)	0.770
D dimer (0–0.5 mg/L)	2.67 (0.5–15)	3.26 (0.2–35)	5.5 (0.3–30)	0.002*
WBCs (4–10 × 10^3/μL)	11.2 (3.4–50.6)	10.3 (2.3–29.4)	12.1 (4.9–27.5)	0.0088*
Lymphocytes(1–3 × 10^3/μL)	1.9 (0.2–12.2)	1.49 (0.3–10)	1.2 (0.7–2.9)	0.634
Neutrophils(1.8–7.7 × 10^3/μL)	8.1 (1.6–44.5)	7.8 (1–26.5)	9.9 (3.5–22.8)	0.340
Platelets (150–410 × 10^3/μL)	262.7 (77–759)	292.6 (53–1047)	351.6 (142–972)	0.540
Hemoglobin (11–16 g/dL)	12.2 (3.4–16.3)	12.1 (3.4–17.2)	11.9 (9–15)	0.269
RBCs (4–6 × 10^6/μL)	4.3 (1.6–5.5)	4.38 (2–6)	4.4 (3.2–5.7)	0.976
PT	13.5 (11.1–25.3)	14.3 (10–45)	14.27 (12–21)	0.913
AST (5–41 U/L)	45.3 (10–208)	78.4 (7–4399)	143.3 (24–1182)	0.946
ALT (5–41 U/L)	35.9 (7–167)	42.5 (2–1825)	142.42 (8–1209)	0.060
ALP (50–140 U/L)	101.5 (27–443)	112.8 (4–478)	145.25 (47–403)	0.279
Serum creatinine (44–116/μmol/L)	112 (102–465)	117.25 (38–415)	124.25 (62–406)	0.341
Serum urea (2.76–8.07 mmol/L)	8.4 (2–40)	9.18 (1.8–41.7)	9.4 (3.9–32.4)	0.341
Lactate dehydrogenase (100–190 U/L)	405 (90–1515)	431.86 (103–1376)	515.75 (227–1849)	0.409
Serum albumin (34–35G/L)	33.7 (21.6–45–6)	33.42 (16–50)	32.7 (22.1–41.3)	0.582
Total protein (64–86 G/L)	70.145 (53–88.4)	69.13 (30–89)	66.9 (52.3–82)	0.146
Sodium (135–145 mmol/L)	139.2 (122–152)	135.49 (132–151)	136 (123–145)	0.169
Amylase (28–100 U/L)	95.6 (10–801)	67.52 (10–437)	109.3 (23–485)	0.298
**Renal involvement**				
Albuminuria(N/%)	38 (69.1)	63 (50)	3 (25)	0.007*
Hematuria(N/%)	40 (72.7)	54 (42.9)	3 (25)	0.002*
AKI(N/%)	33 (60)	34 (27)	0.0 (0)	0.195
Pyuria (N/%)	27 (49.1)	50 (39.7)	2 (16.7)	0.104
Granular casts in urine(N/%)	15 (27.3)	8 (6.3)	1 (8.3)	0.001*
**Admission (days)**	19.1 (7–48)	20.3 (7–43)	26.67 (13–48)	0.530
**Outcome**				
Living (N/%)	17 (30.9)	108 (85.7)	11 (91.7)	0.001*
Dead (N/%)	38 (69.1)	18 (14.3)	1 (8.3)	0.001*

Significance was set at *P*-value < 0.05.

### Hematuria and proteinuria

Dipstick examinations of urine samples from all participants showed that 104 (53.9%) and 98 (50.7%) patients developed proteinuria and hematuria, respectively, after admission. A high prevalence of hematuria was noticed among hypertensive and diabetic male patients with COVID-19(with statistical significance).

Proteinuria and hematuria were associated with a high probability of ICU admission among the patients with COVID-19. Patients with hematuria had a higher probability of ICU admission compared with those without hematuria (OR, 3.789; 95% CI, 1.913–7.505; *P =* 0.003). Conversely, patients with proteinuria had a higher incidence of ICU admission compared with those without proteinuria (OR, 2.493; 95% CI, 1.25–4.72; *P =* 0.001). Moreover, critical cases had a higher prevalence of both proteinuria and hematuria compared with moderate and severs cases (Table 1).

### Microscopic examination

Microscopic examination of urine samples from all participants showed variable results. Sixty-five (33.7%) of them had pus cells in their urine sediment. No difference was recognized between male and female patients. The culture of the urine of patients with pyuria showed that 47 (72.3%) of them had no growth of organisms in culture where as the remaining 18 (27.7%) patients showed growth of various organisms (Table 2).

**Table 2:** Results of the culture of the urine of patients with COVID-19 and pyuria.

**Organism**	**Frequency (N)**	**Percentage (%)**
Negative	47	88.7
*Acinetobacter*	1	0.5
*Candida* (non-albicans)	8	5.7
*E*. *Coli*	1	1.0
*Enterococcus*	4	2.1
*Klebsiella*	1	0.5
*Pseudomonas*	2	1
*Staphylococcus*	1	0.5
Total	65	100.0

In contrast, 24 (12.4%) of all participants showed brown muddy granular casts on urine examination. No difference was found between male and female patients. Diabetes mellitus was a significant risk factor for the presence of casts in urine. No other variables were significantly associated with sterile pyuria, urinary tract infection, or the presence of casts in urine. Both pyuria and urine casts were more prevalent in critical cases (Table 1).

### Acute kidney injury

It is well known that AKI may complicate SARS-COV-2 infection and may be a life-threatening condition. Therefore, the serum creatinine level was monitored strictly in those patients. During the observation period, 48 (23.3%) patients developed AKI according to the criteria defined in the present work. The mean time to the occurrence of AKI from symptom onset was 11 days. Patients in the AKI group were older than patients with no AKI (*P*< 0.001). Moreover, diabetes and hypertension were statistically significant risk factors for the development of AKI, and 73.3% of patients in the AKI group were critical cases. AKI was more prevalent among patients admitted to the ICU (OR, 14.946; 95% CI, 6.7–33.143; *P =* 0.001). Inflammatory markers, including CRP and ESR, were higher in the AKI group. In addition, blood cell and neutrophils counts were higher among AKI cases. The clinical characteristics and laboratory findings of patients who developed AKI are shown in Table 3.

**Table 3:** Clinical and laboratory data in relation to AKI in patients with COVID-19.

**Variable**	**AKI**	**Non AKI**	***P*-value**
**Clinical characteristics**			
**Age (years)**	68.7 (21–95)	61.8 (18–95)	0.074*
**Male patients (n/%)**	28 (62.2)	89 (60.1)	0.001*
**Diabetes (N/%)**	21 (46.7)	74 (50)	0.003*
**Hypertension (N/%)**	18 (40)	47 (31.8)	0.001*
**Clinical presentation**			
Fever and cough (N/%)	19 (42.2)	45 (30.4)	0.041*
Fever (N/%)	3 (6.7)	34 (23)	0.033*
Shortness of breath (N/%)	23 (51.1)	69 (46.6)	0.001*
**Cardiac diseases (N/%)**	1 (2.2)	11 (7.4)	**0.183**
**Severity**			
**Critical**	33 (73.3)	22 (14.9)	0.003*
**Severe**	12 (26.7)	12 (8.1)	0.001*
**Moderate**	0 (0)	114 (77)	0.003*
**ICU admission**			
**Yes**	33 (73.3)	22 (14.9)	0.001*
**No**	12 (26.7)	126 (85.10)	0.001*
**Admission (days)**	19.7 (7–45)	20.3 (7–48)	**0.530**
**Laboratory data**			
**CRP (0–3.3 mg/L)**	87.8 (14–211)	69.3 (1–285)	**0.348**
**ESR (0–29)**	72.7 (23–111)	69.3 (16–120)	**0.111**
**D dimer (0–0.5 mg/L)**	2.7 (0.2–15.2)	3.4 (0–35)	0.002*
**WBCs (4–10 × 10^3/μL)**	11.3 (2.7–50.6)	10.4 (2.5–27.5)	**0.298**
**Lymphocytes(1–3 × 10^3/μL)**	1.5 (0.2–6.8)	1.5 (0.3–12.2)	**0.548**
**Neutrophils (1.8–7.7 × 10^3/μL)**	8.4 (1.5–44.5)	7.9 (1–2.4)	**0.481**
**Platelets (150–410 × 10^3/μL)**	217.8 (77–759)	287.6 (53–1074)	**0.392**
**Hemoglobin(11–16 g/dL)**	12 (6.9–15.7)	12.0 (3.4–17.2)	**0.184**
**RBCs (4–6 × 10^6/μL)**	4.4 (2.5–5.7)	4.3 (2–6)	0.007*
**PT**	13.9 (10.5–22.5)	14.3 (10–45)	**0.521**
**AST (5–41 U/L)**	41.6 (14–208)	81.9 (8–4933)	**0.334**
**ALT (5–41 U/L)**	34.6 (6–167)	50.36 (2–1825)	**0.381**
**ALP (50–140 U/L)**	94.7 (27–313)	115.8 (4–471)	**0.672**
**Serum creatinine (44–116/μmol/L)**	221.5 (120–342)	91.43 (34–112)	0.002*
**Serum urea (2.76–8.07 mmol/L)**	18.6 (11.8–39.9)	8.997 (1.8–41.7)	0.004*
**Lactate dehydrogenase (100–190 U/L)**	369.89 (147–815)	447.8 (90–7054)	**0.393**
**Serum albumin (34–35G/L)**	33.9 (18–50.1)	33.35 (16–47)	**0.131**
**Total protein (64–86 G/L)**	70.9 (40–80.6)	69.06 (30–89)	**0.64**
**Sodium (135–145 mmol/L)**	134 (11–151)	136.2 (136–150)	**0.205**
**Amylase (28–100 U/L)**	80.2 (10–443)	77.51 (10–801)	**0.345**
**Outcome**			
**Living (N/%)**	12 (24.4)	124 (83.8)	0.001*
**Dead (N/%)**	33 (75.6)	24 (16.2)	0.003*

^*^Significance was set at *P*< 0.05.

In the present study, hematuria and proteinuria were significant risk factors for AKI. Patients with hematuria (OR, 2.4; 95% CI, 1.2–4.9; *P =* 0.001) and proteinuria (OR, 1.56; 95% CI, 1.91–7.50; *P =* 0.003) had a higher incidence of AKI compared with those without hematuria or proteinuria (Table 4).

**Table 4:** Risk factors for the development of AKI among patients with COVID-19.

**Variable**	**AKI**	**Non AKI**	***P*-value**
**Albuminuria(N/%)**	28(62.2)	76(51.4)	0.007*
**Hematuria(N/%)**	30(66.7)	67(45.2)	0.009*
**Pyuria (N/%)**	21(46.7)	58(39.2)	0.004*
**Fine granular casts in urine(N/%)**	10(22.2)	14(9.5)	0.006*

^*^Significance was set at *P*< 0.05

### Mortality

The patients included in the present study were followed-up until discharge (either due to recovery or death). Fifty-seven patients (29.5%) died during the observation period. Thirty (52.6%) cases had diabetes and 21 (36.8%) cases had hypertension (with statistical significance). Patients admitted to the ICU were at a higher risk of death (OR, 13.111; 95% CI, 6.249–27.511; *P =* 0.003). Patients with hematuria (OR, 8.084; 95% CI, 3.756–17.397; *P* = 0.002), proteinuria (OR, 2.764; 95% CI, 1.368–5.121; *P =* 0.003), and AKI (OR, 14.208; 95% CI, 6.434–31.375; *P =* 0.003) were at a higher risk of death than were patients without those findings. μLA multivariate analysis of risk factors for mortality using COX regression showed that age and hypertension, in addition to renal manifestations in the form of AKI, hematuria, proteinuria, pyuria, and presence of casts, were significant risk factors for mortality in patients with COVID-19 (Table 5).

**Table 5:** Multivariate COX regression analysis of risk factors for mortality among the patients with COVID-19 included in the present study.

Variable	Hazard Ratio	95.0% Confidence Interval	*P*-value
Age	1.026	1.000–1.053	0.054
Gender	1.726	0.871–3.417	0.118
ICU admission	0.264	0.014–4.938	0.373
Severity	1.036	0.092–11.690	0.977
Pyuria	0.534	0.214–1.334	0.002*
Hematuria	0.431	0.189–.987	0.046*
Albuminuria	0.695	0.313–1.542	0.001*
Casts in urine	0.930	0.377–2.294	0.004*
Hypertension	1.521	0.572–4.047	0.004*
Diabetes mellitus	1.185	0.512–2.745	0.691
Cardiac diseases	6.305	0.676–58.844	0.106
AKI	0.222	0.093–.529	0.001*
WBCS	1.106	0.795–1.541	0.549
LYMPH	0.711	0.400–1.262	0.244
NEUTRO	0.906	0.631–1.301	0.594
HB	0.944	0.680–1.311	0.731
RBCS	1.348	0.456–3.991	0.589
PLATLETS	0.999	0.995–1.002	0.420
PT	1.309	0.965–1.776	0.083
AST	0.999	0.991–1.006	0.717
ALT	1.001	0.987–1.016	0.853
ALP	1.008	1.001–1.014	0.029*
S.UREA	0.879	0.793–0.975	0.014*
S.CREATININE	1.007	1.002–1.011	0.003*
LDH	1.000	0.998–1.001	0.711
S.SODIUM	0.978	0.949–1.009	0.163
S.ALBUMIN	0.943	0.868–1.023	0.159
T.PROTEIN	1.016	0.960–1.074	0.590
AMYLASE	0.999	0.995–1.002	0.451
CRP	1.000	0.994–1.006	0.930
ESR	1.001	0.988–1.014	0.904
D dimer	0.919	0.818–1.033	0.158

^*^Significance was set at *P*< 0.05.

**Fig 1:**
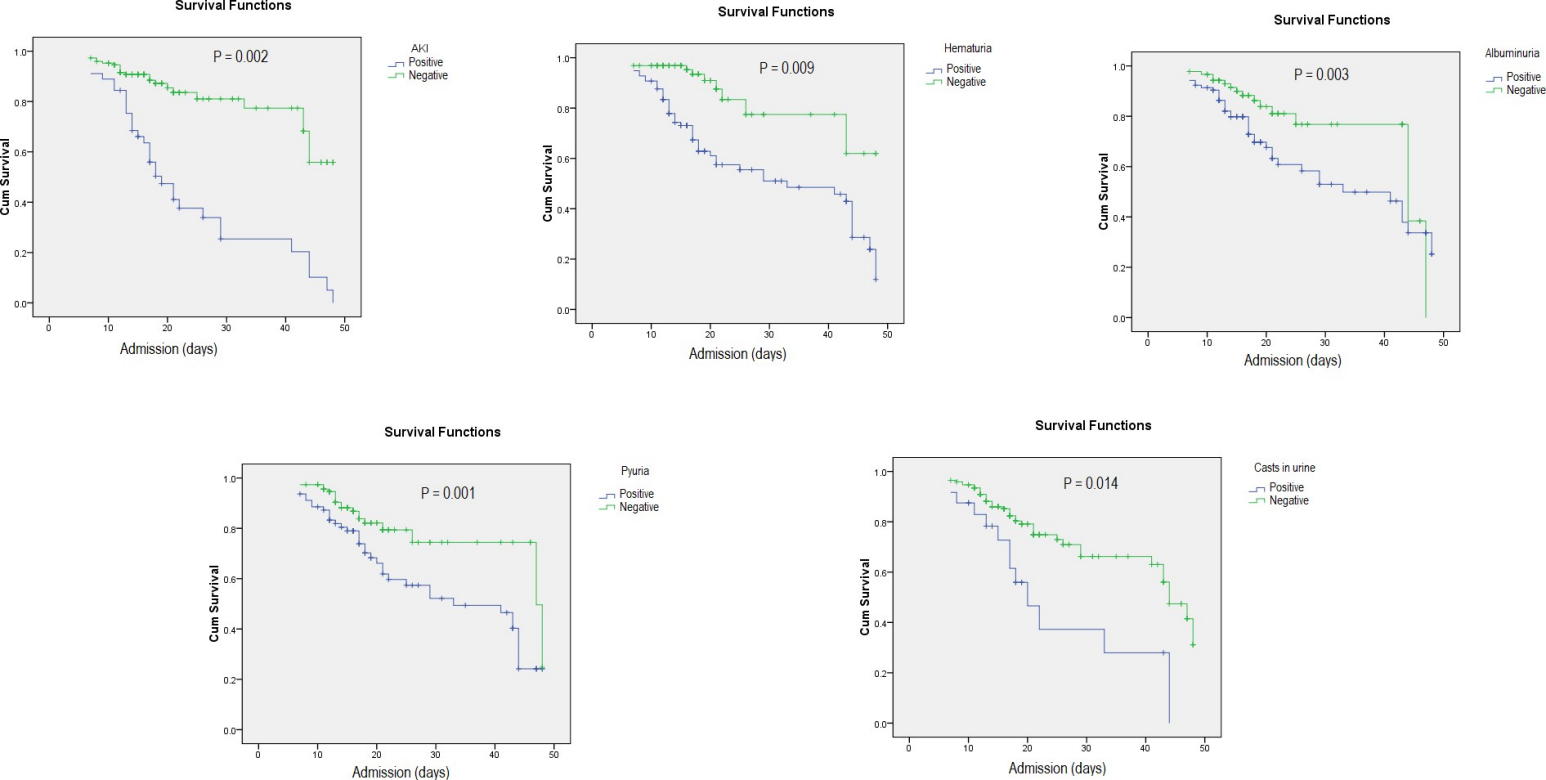
Renal involvement is associated with an increased risk of death among hospitalized patients with COVID-19. A Kaplan–Meier analysis of the effect of different manifestations of renal involvement on case fatality in hospitalized patients with COVID-19 showed that renal involvement is associated with an increased risk of death (*P*-value< 0.05 for all variables).

Moreover, a univariate analysis using the renal variables as risk factors for mortality in patients with COVID-19 showed that all of these parameters were associated with an increased risk of death in the patients who developed those manifestations (Fig 1).

## Discussion

COVID-19 has been reported mainly as a serious acute respiratory syndrome caused by alveolar and interstitial pneumonia. Although the main organ system involved in the manifestation of the disease is the pulmonary system, other organs, such as the gastrointestinal tract and the renal and nervous systems, are also involved in infected patients. Recent studies indicate that renal disorder is prevalent in patients with COVID-19 [29, 35]. The present research aimed to determine the involvement of the renal system in these patients and its importance as a prognostic factor in monitoring disease outcome. To the best of our knowledge, this was the first study of the prevalence and relationship of hematuria, proteinuria, and AKI with patient outcomes among hospitalized patients with COVID-19 in Saudi Arabia.

The present study included 193 COVID-19-positive patients. Most of them were elderly males, which is consistent with previous studies [29, 30, 35–39]. The high disease prevalence among males can be explained by the suppression of the immune system by the male sex hormone testosterone [40]. It is well documented that hypo-androgenism is associated with increased inflammatory cytokines, antibody titers, CD4/CD8 ratios, and natural killer cells, as well as a decrease in regulatory T cells [40]. Reduced immunity and ahigh prevalence of associated co-morbidities (mainly diabetes and hypertension) may explain the high prevalence of the disease among elderly patients, which is in accordance with other reports [41].

Variable findings regarding renal system affection in COVID-19 were reported. Regarding the dipstick examination of the urine of all participants, proteinuria and hematuria were reported in 53.9% and 22.2% of the patients, respectively. We noted that proteinuria and hematuria were associated with ICU admission. Patients with hematuria had a 3.7 times higher risk of ICU admission, where as those with proteinuria had a 2.6 times higher risk of ICU admission. These results are in agreement with previous reports [22, 24]. Cheng et al. [24] reported a prevalence of 43.9% and 26.7% of proteinuria and hematuria, respectively, in a prospective cohort study of 701 patients with COVID-19 in Wuhan, which is similar to the results reported by Hirsch et al. in a cohort of 646 patients from New York City, where he found a prevalence of 42.1% of proteinuria [23]. Pei et al. reported a prevalence of 65.8% for proteinuria and 41.7% for hematuria among 333 patients with COVID-19 [22].

In the present work, 45 patients (23.3%) developed AKI. Patients with hematuria had a 2.4 times higher risk of AKI, whereas patients with proteinuria had a 1.7 times higher risk of AKI than did those without hematuria or proteinuria, which is accordance with previous reports [18, 22, 24]. Chan et al. reported that 84% of patients with proteinuria and 81% of patients with hematuria developed AKI [42]. Another study reported by Chaudhri et al. revealed that patients with proteinuria hada4.7 times higher risk of development of AKI [18]. Pei et al. estimated that, among the 333 patients examined, those with AKI had a higher incidence rate of proteinuria (88.6% vs.63.1%) and hematuria (60% vs.41.7%) compared with the non-AKI group [22]. Studies of patients with COVID-19 reported an incidence of AKI ranging from 5% to 29% [24, 26–31, 38, 43]. Cheng et al. [24] reported a lower rate of AKI of 5.1% among701 patients from Wuhan, China. This may be attributed to lower rates of co-morbidities, such as diabetes and hypertension, and lower prevalence of respiratory disease severity, as reported by the authors (only 13.4% of patients were critically ill). A greater incidence of AKI was reported in US hospitals, which may be attributed to the differences in the baseline characteristics of patients [23]. In the present wok, 46.7% of the patients in the AKI group had diabetes and 40% had hypertension. Hypertension was a significant risk factor for the development of AKI in the present study, which is in accordance with other studies of AKI in COVID-19 [23]. This may be explained by the fact that associated vascular diseases might contribute to impaired auto-regulation in the kidney, thereby placing hypertensive patients at a higher risk of AKI from the vascular and hemodynamic abnormalities noted in COVID-19 [18].

In the present study, we reported pyuria in 65 patients, the majority of whom showed no growth on culture. Pyuria in this case was sterile, which may be caused by viral cystitis [44]. It is well known that SARS-COV-2 can be detected in the urine [45, 46]. In addition, SARS-CoV-2 infection may cause ladder dysfunction (Mumm et al. 2020). Dhar et al. reported that COVID-19-positive patients developed *de novo* urinary symptoms without urinary tract infection, as assessed by urine culture [47]. Mumm et al. first reported an increased frequency of micturition among patients with COVID-19 [47, 48].

During the observation period, 57 patients died. We noticed that patients with COVID-19who developed hematuria (OR, 8.084; 95% CI, 3.756–17.397; *P* = 0.002), proteinuria (OR, 2.764; 95% CI, 1.368–5.121; *P =* 0.003), and AKI (OR, 14.208; 95% CI, 6.434–31.375; *P =* 0.003) were at a higher risk of death than were other patients (with statistical significance). Our data are in accordance with the results of other studies [18, 23, 24]. Chaudhri et al. studied 321 patients in New York; they found that patients with proteinuria and AKI had a higher risk of death compared with other patients [18]. In a Chinese study of 701 patients hospitalized for COVID-19, Cheng et al. reported that proteinuria and hematuria were associated with an increased risk of in-hospital death after adjusting for all other variables [24]. Pei et al. analyzed 333 Chinese patients hospitalized for COVID-19 and observed that those with renal involvement (hematuria, proteinuria, or AKI) had higher rates of mortality than did those without these conditions [22].

## Conclusion

Hematuria, proteinuria, and AKI are highly prevalent in patients with COVID-19 and are associated with adverse outcomes in hospitalized patients with COVID-19. Patients who develop renal system involvement in the form of the aforementioned manifestations are at higher risk of ICU admission, AKI, and mortality than are those without renal involvement. To prevent fatality in such cases, we suggest strict monitoring of kidney function in patients with COVID-19, regardless of the past history of kidney disease. Therefore, clinicians can consider any potential interventions to protect kidney function at the early stage of the disease. Further studies are required to investigate the etiologies of these kidney abnormalities.

The present study was performed at a single center in addition to having an observational retrospective design, which limited the availability of some data that could explain the etiology of AKI, to propose specific prevention or treatment measures. Therefore, additional more detailed studies of the clinical data of patients are required to help explain the pathophysiology of AKI in patients with COVID-19.

## References

[1] Lu H. Drug treatment options for the 2019-new coronavirus (2019-nCoV). Biosci Trends. 2020;14: 69–71. doi: 10.5582/bst.2020.01020 31996494

[2] Wang W, Tang J, Wei F. Updated understanding of the outbreak of 2019 novel coronavirus (2019-nCoV) in Wuhan, China. J Med Virol. 2020;92: 441–447. doi: 10.1002/jmv.25689 31994742 PMC7167192

[3] Li W, Moore MJ, Vasilieva N, Sui J, Wong SK, Berne MA, et al. Angiotensin-converting enzyme 2 is a functional receptor for the SARS coronavirus. Nature. 2003;426: 450–454. doi: 10.1038/nature02145 14647384 PMC7095016

[4] Chen Y, Guo Y, Pan Y, Zhao ZJ. Structure analysis of the receptor binding of 2019-nCoV. Biochem Biophys Res Commun. 2020;525: 135–140. doi: 10.1016/j.bbrc.2020.02.071 32081428 PMC7092824

[5] Zou X, Chen K, Zou J, Han P, Hao J, Han Z. Single-cell RNA-seq data analysis on the receptor ACE2 expression reveals the potential risk of different human organs vulnerable to 2019-nCoV infection. Front Med. 2020;14: 185–192. doi: 10.1007/s11684-020-0754-0 32170560 PMC7088738

[6] Gavriatopoulou M, Korompoki E, Fotiou D, Ntanasis-Stathopoulos I, Psaltopoulou T, Kastritis E, et al. Organ-specific manifestations of COVID-19 infection. Clin Exp Med. 2020;20: 493–506. doi: 10.1007/s10238-020-00648-x 32720223 PMC7383117

[7] Richardson S, Hirsch JS, Narasimhan M, Crawford JM, McGinn T, Davidson KW, et al. Presenting Characteristics, Comorbidities, and Outcomes Among 5700 Patients Hospitalized With COVID-19 in the New York City Area. JAMA. 2020;323: 2052–2059. doi: 10.1001/jama.2020.6775 32320003 PMC7177629

[8] Guan W-J, Ni Z-Y, Hu Y, Liang W-H, Ou C-Q, He J-X, et al. Clinical Characteristics of Coronavirus Disease 2019 in China. N Engl J Med. 2020;382: 1708–1720. doi: 10.1056/NEJMoa2002032 32109013 PMC7092819

[9] Grasselli G, Zangrillo A, Zanella A, Antonelli M, Cabrini L, Castelli A, et al. Baseline Characteristics and Outcomes of 1591 Patients Infected With SARS-CoV-2 Admitted to ICUs of the Lombardy Region, Italy. JAMA. 2020;323: 1574–1581. doi: 10.1001/jama.2020.5394 32250385 PMC7136855

[10] Analysis on 54 Mortality Cases of Coronavirus Disease 2019 in the Republic of Korea from January 19 to March 10, 2020. J Korean Med Sci. 2020;35: e132. doi: 10.3346/jkms.2020.35.e132 32233161 PMC7105509

[11] Yan Y, Yang Y, Wang F, Ren H, Zhang S, Shi X, et al. Clinical characteristics and outcomes of patients with severe covid-19 with diabetes. BMJ open diabetes Res care. 2020;8. doi: 10.1136/bmjdrc-2020-001343 32345579 PMC7222577

[12] Chaudhri I, Moffitt R, Taub E, Annadi RR, Hoai M, Bolotova O, et al. Association of Proteinuria and Hematuria with Acute Kidney Injury and Mortality in Hospitalized Patients with COVID-19. Kidney Blood Press Res. 2020;11716: 1018–1032. doi: 10.1159/000511946 33171466

[13] Li J-W, Han T-W, Woodward M, Anderson CS, Zhou H, Chen Y-D, et al. The impact of 2019 novel coronavirus on heart injury: A Systematic review and Meta-analysis. Prog Cardiovasc Dis. 2020;63: 518–524. doi: 10.1016/j.pcad.2020.04.008 32305557 PMC7160645

[14] Wilson MP, Jack AS. Coronavirus disease 2019 (COVID-19) in neurology and neurosurgery: A scoping review of the early literature. Clin Neurol Neurosurg. 2020;193. doi: 10.1016/j.clineuro.2020.105866 32389893 PMC7179494

[15] Fogarty H, Townsend L, Ni Cheallaigh C, Bergin C, Martin-Loeches I, Browne P, et al. COVID19 coagulopathy in Caucasian patients. Br J Haematol. 2020;189: 1044–1049. doi: 10.1111/bjh.16749 32330308 PMC7264579

[16] Bouaziz JD, Duong TA, Jachiet M, Velter C, Lestang P, Cassius C, et al. Vascular skin symptoms in COVID-19: a French observational study. Journal of the European Academy of Dermatology and Venereology: JEADV. 2020. pp. e451–e452. doi: 10.1111/jdv.16544 32339344 PMC7267662

[17] Fujimaru T, Shimada K, Hamada T, Watanabe K, Ito Y, Nagahama M, et al. Development of acute kidney injury with massive granular casts and microscopic hematuria in patients with COVID-19: two case presentations with literature review. Ren Replace Ther. 2020;6. doi: 10.1186/s41100-020-00308-6 33510902 PMC7716112

[18] Chaudhri I, Moffitt R, Taub E, Annadi RR, Hoai M, Bolotova O, et al. Association of Proteinuria and Hematuria with Acute Kidney Injury and Mortality in Hospitalized Patients with COVID-19. Kidney Blood Press Res. 2020;11716: 1018–1032. doi: 10.1159/000511946 33171466

[19] Gross O, Moerer O, Weber M, Huber TB, Scheithauer S. COVID-19-associated nephritis: early warning for disease severity and complications? Lancet (London, England). 2020. pp. e87–e88. doi: 10.1016/S0140-6736(20)31041-2 32423587 PMC7202828

[20] Hernandez-Arroyo CF, Varghese V, Mohamed MMB, Velez JCQ. Urinary Sediment Microscopy in Acute Kidney Injury Associated with COVID-19. Kidney360. 2020;1: 819–823. doi: 10.34067/kid.0003352020 PMC8815753 35372960

[21] Liu R, Ma Q, Han H, Su H, Liu F, Wu K, et al. The value of urine biochemical parameters in the prediction of the severity of coronavirus disease 2019. Clin Chem Lab Med. 2020;58: 1121–1124. doi: 10.1515/cclm-2020-0220 32286242

[22] Pei G, Zhang Z, Peng J, Liu L, Zhang C, Yu C, et al. Renal Involvement and Early Prognosis in Patients with COVID-19 Pneumonia. J Am Soc Nephrol. 2020;31: 1157–1165. doi: 10.1681/ASN.2020030276 32345702 PMC7269350

[23] Hirsch JS, Ng JH, Ross DW, Sharma P, Shah HH, Barnett RL, et al. Acute kidney injury in patients hospitalized with COVID-19. Kidney Int. 2020;98: 209–218. doi: 10.1016/j.kint.2020.05.006 32416116 PMC7229463

[24] Cheng Y, Luo R, Wang K, Zhang M, Wang Z, Dong L, et al. Kidney disease is associated with in-hospital death of patients with COVID-19. Kidney Int. 2020;97: 829–838. doi: 10.1016/j.kint.2020.03.005 32247631 PMC7110296

[25] Lv W, Wu M, Ren Y, Zeng N, Deng P, Zeng H, et al. Coronavirus Disease 2019: Coronaviruses and Kidney Injury. J Urol. 2020;204: 918–925. doi: 10.1097/JU.0000000000001289 32693711

[26] Castro NM, Rodrigues WJ, Freitas DM, Muniz A, Oliveira P, Carvalho EM. Urinary symptoms associated with human T-cell lymphotropic virus type I infection: evidence of urinary manifestations in large group of HTLV-I carriers. Urology. 2007;69: 813–818. doi: 10.1016/j.urology.2007.01.052 17482910

[27] Chen T, Wu D, Chen H, Yan W, Yang D, Chen G, et al. Clinical characteristics of 113 deceased patients with coronavirus disease 2019: retrospective study. BMJ. 2020;368. doi: 10.1136/bmj.m1091 32217556 PMC7190011

[28] Diao B, Wang C, Wang R, Feng Z, Tan Y, Wang H, et al. Human Kidney is a Target for Novel Severe Acute Respiratory Syndrome Coronavirus 2 (SARS-CoV-2) Infection. 2020;2. doi: 10.1101/2020.03.04.20031120 PMC8096808 33947851

[29] Huang C, Wang Y, Li X, Ren L, Zhao J, Hu Y, et al. Clinical features of patients infected with 2019 novel coronavirus in Wuhan, China. Lancet. 2020;395: 497–506. doi: 10.1016/S0140-6736(20)30183-5 31986264 PMC7159299

[30] Wang D, Hu B, Hu C, Zhu F, Liu X, Zhang J, et al. Clinical Characteristics of 138 Hospitalized Patients with 2019 Novel Coronavirus-Infected Pneumonia in Wuhan, China. JAMA—J Am Med Assoc. 2020;323: 1061–1069. doi: 10.1001/jama.2020.1585 32031570 PMC7042881

[31] Zhou F, Yu T, Du R, Fan G, Liu Y, Liu Z, et al. Clinical course and risk factors for mortality of adult inpatients with COVID-19 in Wuhan, China: a retrospective cohort study. Lancet. 2020;395: 1054–1062. doi: 10.1016/S0140-6736(20)30566-3 32171076 PMC7270627

[32] Ahmadian E, Hosseiniyan Khatibi SM, Razi Soofiyani S, Abediazar S, Shoja MM, Ardalan M, et al. Covid-19 and kidney injury: Pathophysiology and molecular mechanisms. Rev Med Virol. 2020; 1–13. doi: 10.1002/rmv.2176 33022818 PMC7646060

[33] Khwaja A. KDIGO clinical practice guidelines for acute kidney injury. Nephron—Clin Pract. 2012;120: 179–184. doi: 10.1159/000339789 22890468

[34] Horan TC, Andrus M, Dudeck MA. CDC/NHSN surveillance definition of health care-associated infection and criteria for specific types of infections in the acute care setting. Am J Infect Control. 2008;36: 309–332. doi: 10.1016/j.ajic.2008.03.002 18538699

[35] Peiris JSM, Chu CM, Cheng VCC, Chan KS, Hung IFN, Poon LLM, et al. Clinical progression and viral load in a community outbreak of coronavirus-associated SARS pneumonia: a prospective study. Lancet (London, England). 2003;361: 1767–1772. doi: 10.1016/s0140-6736(03)13412-5 12781535 PMC7112410

[36] Zhang J-J, Dong X, Cao Y-Y, Yuan Y-D, Yang Y-B, Yan Y-Q, et al. Clinical characteristics of 140 patients infected with SARS-CoV-2 in Wuhan, China. Allergy. 2020;75: 1730–1741. doi: 10.1111/all.14238 32077115

[37] Li Q, Guan X, Wu P, Wang X, Zhou L, Tong Y, et al. Early Transmission Dynamics in Wuhan, China, of Novel Coronavirus–Infected Pneumonia. N Engl J Med. 2020;382: 1199–1207. doi: 10.1056/NEJMoa2001316 31995857 PMC7121484

[38] Chen N, Zhou M, Dong X, Qu J, Gong F, Han Y, et al. Epidemiological and clinical characteristics of 99 cases of 2019 novel coronavirus pneumonia in Wuhan, China: a descriptive study. Lancet. 2020;395: 507–513. doi: 10.1016/S0140-6736(20)30211-7 32007143 PMC7135076

[39] Yang X, Yu Y, Xu J, Shu H, Xia J, Liu H, et al. Clinical course and outcomes of critically ill patients with SARS-CoV-2 pneumonia in Wuhan, China: a single-centered, retrospective, observational study. Lancet Respir Med. 2020;8: 475–481. doi: 10.1016/S2213-2600(20)30079-5 32105632 PMC7102538

[40] Klein SL, Flanagan KL. Sex differences in immune responses. Nat Rev Immunol. 2016;16: 626–638. doi: 10.1038/nri.2016.90 27546235

[41] Meng Y, Wu P, Lu W, Liu K, Ma K, Huang L, et al. Sex-specific clinical characteristics and prognosis of coronavirus disease-19 infection in Wuhan, China: A retrospective study of 168 severe patients. PLoS Pathog. 2020;16: 1–13. doi: 10.1371/journal.ppat.1008520 32343745 PMC7209966

[42] Chan L, Chaudhary K, Saha A, Chauhan K, Vaid A, Zhao S, et al. AKI in hospitalized patients with COVID-19. J Am Soc Nephrol. 2021;32: 151–160. doi: 10.1681/ASN.2020050615 32883700 PMC7894657

[43] Guan W, Ni Z, Hu Y, Liang W, Ou C, He J, et al. Clinical Characteristics of Coronavirus Disease 2019 in China. N Engl J Med. 2020;382: 1708–1720. doi: 10.1056/NEJMoa2002032 32109013 PMC7092819

[44] Cao Hongmei, Yu Rui, Tao Yi, Nikolic Dejan and RB van B. 基因的改变NIH Public Access. Bone. 2005;23: 1–7. doi: 10.1016/j.juro.2010.12.043.HIV

[45] Souza SP de, Silveira MAD, Souza BS de F, Nonaka CKV, Melo E de, Cabral JB, et al. Evaluation of Urine SARS-COV-2 RT-PCR as a predictor of Acute Kidney Injury and disease severity in critical COVID-19 patients. medRxiv. 2021. Available: https://medrxiv.org/cgi/content/short/2021.01.13.21249576 10.1177/03000605211015555 PMC8127770 33990155

[46] Frithiof R, Bergqvist A, Järhult JD, Lipcsey M, Hultström M. Presence of SARS-CoV-2 in urine is rare and not associated with acute kidney injury in critically ill COVID-19 patients. Crit Care. 2020;24: 4–6. doi: 10.1186/s13054-019-2709-x 32993742 PMC7523248

[47] Dhar N, Dhar S, Timar R, Lucas S. Letter to the Editor De Novo Urinary Symptoms Associated With COVID-19: 2020;12: 681–682. 10.14740/jocmr4294 PMC7524562 33029276

[48] Mumm J, Osterman A, Ruzicka M, Stihl C, Stief C, Staehler M, et al. Since January 2020 Elsevier has created a COVID-19 resource centre with free information in English and Mandarin on the novel coronavirus COVID- 19. The COVID-19 resource centre is hosted on Elsevier Connect, the company ‘ s public news and information. 2020.

